# Hypoparathyroidism in a Child with MELAS Syndrome: A Case Report of Severe Lactic Acidosis and Symmetrical Bilateral Basal Ganglia Calcification

**DOI:** 10.5812/ijem-161585

**Published:** 2025-04-30

**Authors:** Ling Wang, Tingting Zhou, Xiaosong Bu, Daoxiang Pan, Xiaojing Liu

**Affiliations:** 1 The First Affiliated Hospital of Anhui Medical University, Anhui 230000, China

**Keywords:** Mitochondrial Disease, MELAS Syndrome, Hypoparathyroidism, MT-TL1 Gene

## Abstract

**Introduction:**

MELAS syndrome is a mitochondrial disorder typically characterized by brain dysfunction and endocrinopathies, but it rarely presents with hypoparathyroidism (HP).

**Case Presentation:**

Here, we report the case of a child who initially presented with vomiting and convulsions. Blood gas analysis revealed significant hyperlactatemia and hypocalcemia. The child's urinary calcium level was markedly decreased, measured at 0.15 mmol/24h, well below the normal range. A brain CT scan showed symmetrical calcification in the bilateral basal ganglia. Endocrine testing confirmed low parathyroid hormone (PTH) levels. During hospitalization, the child received treatment for recurrent seizures, including midazolam and levetiracetam. One month post-discharge, the child was readmitted due to elevated lactate levels. Genetic testing confirmed the diagnosis of MELAS syndrome, identifying the m.3243A > G mutation in the MT-TL1 gene. Under symptomatic treatment, the child has not experienced any further convulsions and has been regularly followed up at our hospital.

**Conclusions:**

This case underscores the importance of considering MELAS syndrome in patients presenting with hypoparathyroidism. Effective management of epileptic seizures and maintaining an appropriate calcium-to-phosphorus balance are crucial for minimizing brain damage and improving the patient’s prognosis.

## 1. Introduction

Hypoparathyroidism (HP) is an endocrine disorder characterized by the hyposecretion of parathyroid hormone (PTH), with a prevalence of less than 3 cases per 10,000 individuals (1). It is typically marked by hypocalcemia, hyperphosphatemia, and low levels of PTH in the blood, leading to symptoms such as muscle tetany and seizures resembling epilepsy. Additionally, basal ganglia calcification is commonly observed in HP patients, serving as an independent risk factor for seizure-like activity. The development of this calcification is strongly associated with an imbalanced calcium-to-phosphorus ratio. Studies suggest that for every 1% increase in this ratio, the risk of progression decreases by 5% (2).

HP is frequently observed following thyroid surgery, with hereditary HP being the second most common cause. Beyond isolated cases, HP may also occur as part of various syndromes or mitochondrial diseases, such as Kenny-Caffey syndrome or MELAS (Mitochondrial Encephalomyopathy, Lactic Acidosis, and Stroke-like episodes) syndrome; however, idiopathic HP is rare. MELAS syndrome, a mitochondrial disorder caused by mutations in mitochondrial DNA (mtDNA), affects multiple systems, including the central nervous system. Patients typically present with headaches, vomiting, muscle weakness, endocrinopathies, seizures, and stroke-like episodes (3). More than 80% of MELAS patients carry the m.3243A > G mutation in the MT-TL1 (UUR) gene, which encodes the mitochondrial tRNA(Leu) (4). Neuroimaging often reveals multiple brain parenchymal lesions involving the temporal, parietal, and occipital lobes, and basal ganglia calcification (BCG) is present in 30% - 70% of patients (5). These overlapping clinical features often lead to misdiagnosis and delayed treatment.

In this case, we performed whole-exome capture sequencing (mean depth ≥ 90X, covering exonic regions and flanking 5bp sequences) and whole-genome sequencing on the patient, followed by orthogonal validation using Sanger sequencing, to confirm the diagnosis of MELAS syndrome. Fortunately, the patient received an accurate diagnosis and timely intervention.

## 2. Case Presentation

The patient was a 5-year-and-8-month-old Chinese boy, the third-born of three children, with a 12-year-old brother and a 9-year-old sister. He was born at 38 weeks of gestation via spontaneous vaginal delivery, with no history of asphyxia or resuscitation at birth. His mother had three pregnancies (G3P3). The boy achieved developmental milestones as expected: He could hold his head steadily at three months, sit independently at six months, and walk independently at thirteen months.

**Figure 1.**
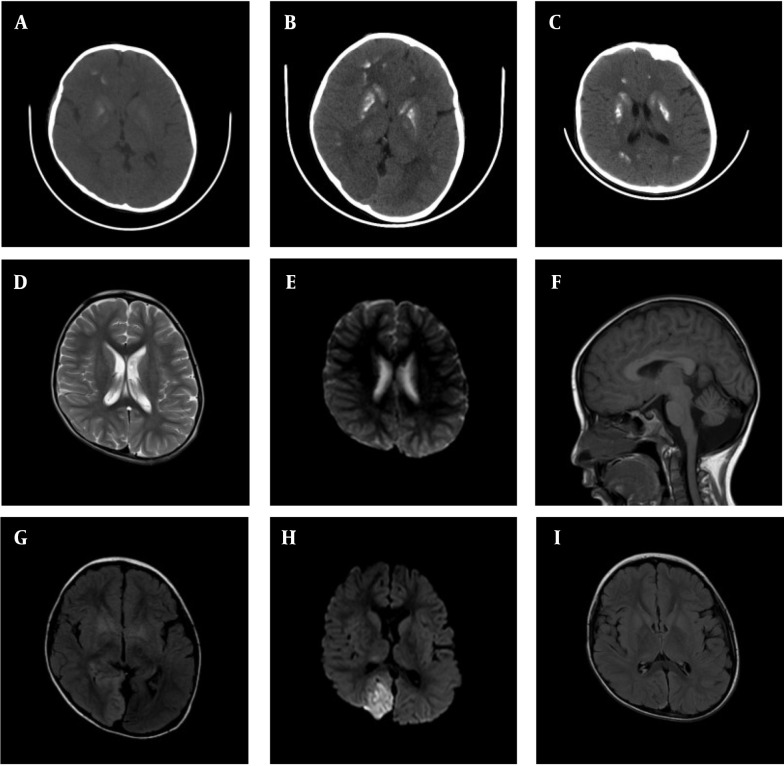
A – C, Brain CT scans obtained one year ago, during the first hospitalization, and at the second hospitalization after the current onset, respectively. Extensive basal ganglia calcification is observed, predominantly affecting the striatum and globus pallidus. Over the past year, the degree of calcification has increased markedly, though recent changes are relatively unremarkable; D and E, head MRI results from the local hospital in 2023, showing no significant abnormal high signals in T2 and DWI sequences; F - H, head MRI results during the first hospitalization in the current onset, showing patchy high-signal areas on T2 and FLAIR sequences, along with high signals on DWI in the right parieto-occipital lobe. Slight cerebellar atrophy is also noted; I, Head MRI results from the second hospitalization during the current onset, showing slight abnormal signals in the right parieto-occipital lobe, with a significant reduction in the extent compared to the previous hospitalization.

Approximately one year ago, he presented to a local hospital with poor appetite and vomiting. Blood gas analysis revealed lactic acidosis, and a CT scan showed multiple intracranial calcifications (Figure 1A). However, his parents declined genetic testing, which was recommended by the local physician at that time. Recently, he presented again with vomiting for two days and a single episode of convulsions, prompting him to seek care at our hospital. Prior to this visit, he had been treated at the local hospital with diazepam, cimetidine, and azithromycin, along with acid-base correction therapy. Despite this, he continued to experience low-grade fever and sleepiness.

The patient’s family members are asymptomatic, although his mother experiences frequent migraine attacks. Physical examination revealed a low hairline, mild left-sided facial asymmetry, and ptosis in both eyelids. There was no significant reduction in limb muscle strength, and no obvious spinal or limb deformities were noted. His height of 104.5 cm was below the expected range for his age (<-2SD), although his language and motor skills appeared normal. Neurological examination showed positive percussion pain over the facial nerve, along with a positive Babinski sign on the left side and a suspected positive Babinski sign on the right.

The patient's initial arterial blood gas analysis revealed the following parameters: pH 7.195 (normal range: 7.35 – 7.45), pCO2 61.2 mmHg (normal range: 32 - 48 mmHg), bicarbonate 23.1 mmol/L (normal range: 22 - 27 mmol/L), calcium (Ca^2+^) 0.97 mmol/L (normal range: 1.15 - 1.33 mmol/L), and lactic acid 6.0 mmol/L (normal range: 1.0 - 1.8 mmol/L). Blood electrolyte analysis showed phosphorus (P) 1.47 mmol/L (normal range: 0.81 - 1.45 mmol/L), and his 25-hydroxyvitamin D level was critically low at 7.8 ng/mL (normal range: > 30 ng/mL). Blood glucose, liver, and kidney functions were within normal limits. The PTH levels varied between 6.62 pg/mL and less than 3 pg/mL (normal range: 10 - 69 pg/mL). A 24-hour urinary calcium test showed 0.15 mmol/24h (normal range: 2.5 - 7.5 mmol/24h), and urinary phosphorus was 7.34 mmol/24h (normal range: 22 - 48 mmol/24h). Cerebrospinal fluid analysis revealed a positive Pandy's reaction. Ultrasonography identified bilateral thyroid cysts, though thyroid function remained normal.

During hospitalization, the patient underwent three electroencephalogram (EEG) tests, all showing consistent findings of prominent, diffuse, asynchronous slow-wave activity localized to the right hemisphere. Epileptiform discharges were observed in the central region during seizures. Cranial MRI (Figure 1) revealed patchy high-signal intensities on T2-weighted and FLAIR sequences, as well as high-signal intensities on DWI in the right parieto-occipital lobe. There was no significant enhancement after contrast administration.

Given the extensive calcification in the basal ganglia, HP was initially diagnosed as Fahr's syndrome. However, considering that Fahr's disease typically does not present with metabolic lactate abnormalities or cortical damage, further investigations were conducted, and symptomatic treatment was initiated. Genetic testing was also recommended, which the parents ultimately agreed to pursue.

The patient's temperature gradually normalized, and vomiting ceased by the fifth day of hospitalization after receiving acid-correction, fluid replacement, and anti-infection therapy. Blood gas analysis showed significant improvement: pH 7.394, pCO2 42.19 mmHg, bicarbonate 25.2 mmol/L, and lactic acid 3.5 mmol/L. Changes in laboratory indicators are shown in Appendix 1 in Supplementary File. Despite the improvement in metabolic parameters, the patient continued to experience recurrent seizures, prompting the initiation of sodium valproate and levetiracetam orally, along with midazolam continuous infusion. The dosage was adjusted according to seizure frequency.

The patient was initially prescribed 300 mg calcium carbonate and 400 IU Vitamin D daily, later increased to twice daily. However, urinary calcium and serum calcium levels showed limited improvement, with the urine calcium test still negative. As a result, intravenous calcium gluconate and oral calcitriol were added to the treatment regimen. Three days later, urinary calcium became positive, and the 25-hydroxyvitamin D level increased to 27.3 ng/mL. Also, PTH levels remained below the detection threshold.

**Figure 2.**
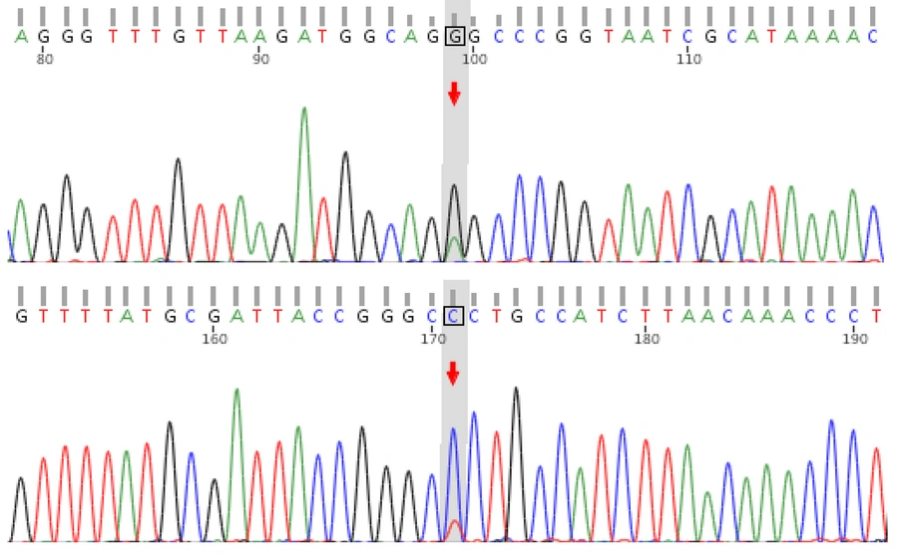
MT-TL1 Sanger Sequencing of the Proband. Electropherogram demonstrating heterozygous m.3243A > G mutation in the proband, confirming mitochondrial heteroplasmy.

Upon discharge, the patient was prescribed levetiracetam 250 mg every 12 hours, 900 mg/d calcium supplements, 0.25 μg/d calcitriol, and 400 IU/d vitamin D, with no further episodes of tetany or vomiting. During the outpatient follow-up one month after discharge, the patient was readmitted due to a reexamination of his blood test, which revealed a lactate level of 6.6 mmol/L. At this point, the family’s genetic test results were also available. Genetic testing identified the m.3243A > G mutation in the MT - TL1 gene in both the patient and his mother (Figure 2), while his brother and sister were wild-type. Combining these genetic findings with the patient’s clinical symptoms, we confirmed a diagnosis of MELAS syndrome.

Unfortunately, due to the family’s wishes, genetic testing could not be performed on other members of the maternal family, preventing the completion of a family pedigree investigation. At this time, the child showed no significant symptoms. The blood gas analysis revealed no abnormalities, except for a lactic acid level of 8.4 mmol/L, and PTH remained below the detection threshold. His vitamin D level was 33.9 ng/mL, and blood electrolytes showed: Calcium (Ca^2+^) 2.47 mmol/L, phosphorus (P) 1.91 mmol/L, and magnesium (Mg^2+^) 0.7 mmol/L. His fasting insulin level was low, at 2.22 mU/L (normal range: 3 - 25 mU/L), though both blood glucose and glycated hemoglobin levels were normal. No abnormalities were detected in the electrocardiogram or echocardiogram. MRI revealed a few abnormal signals in the right parieto-occipital lobe, with the area significantly smaller than in the previous scan (Figure 1I). 

After treatment with levocarnitine, fluid replacement, magnesium supplementation, and other supportive measures, the lactic acid level decreased to 3.5 mmol/L. His treatment regimen was subsequently adjusted. In addition to continuing oral administration of levetiracetam, vitamin D, vitamin B1, vitamin E, calcitriol, and calcium carbonate, the following were added: Levocarnitine 0.5 g three times daily, idebenone 15 mg three times daily, and coenzyme Q10 20 mg twice daily. Additionally, it was recommended that the family switch to "CHILD LIFE" liquid calcium to address the serum magnesium deficiency and maintain the calcium-phosphorus product below 4.4 mmol^2^/L^2^.

Throughout the treatment period, no adverse events were recorded. Prior to discharge, the patient was advised to avoid high-intensity exercise, adjust his diet and lifestyle, and return for regular follow-up appointments.

## 3. Discussion

The m.3243A > G mutation in the MT - TL1 gene is the most commonly identified pathogenic variation in mitochondrial DNA (mtDNA) associated with clinical disease. This mutation has been detected in numerous patients with mitochondrial disorders. Previous studies have shown that it is linked to a range of diseases, primarily mitochondrial encephalopathy, lactic acidosis, MELAS, progressive external ophthalmoplegia (PEO), mitochondrial diabetes, deafness, headaches, and other symptoms. According to the guidelines for variant interpretation from the American College of Medical Genetics and Genomics and the Association for Molecular Pathology (ACMG-AMP), and considering the functional impact, family genetic features, and clinical associations, the m.3243A > G variant is definitively classified as pathogenic.

In this case, the child presented with clinical symptoms including vomiting, diarrhea, altered consciousness, convulsions, short stature, left eyelid ptosis, low PTH levels, HP, and metabolic encephalopathy (6). However, there was no significant damage to the heart or kidneys, and no deafness or hearing impairment was observed. Despite the low fasting insulin level, the patient's blood glucose levels remained normal. Given the clinical presentation and laboratory findings, we were more inclined to diagnose MELAS.

MELAS is a mitochondrial disease caused by mutations in mtDNA, first described in 1984. Its inheritance pattern is primarily maternal, with an age of onset typically between 2 and 40 years. The disease is most commonly associated with single-nucleotide variations in the MT - TL1 (UUR) gene, which encodes the tRNA responsible for transporting leucine in mitochondria. This disruption impairs the translation and synthesis of mitochondrial proteins, leading to mitochondrial dysfunction and inadequate energy production. Organs with high oxygen demand, such as the brain and cardiac muscle, are particularly susceptible. Clinical manifestations of MELAS include stroke-like episodes, encephalopathy (manifesting as seizures and/or dementia), muscle weakness, exercise intolerance, and normal early psychomotor development. Other phenotypes include recurrent headaches, vomiting, hearing impairment, peripheral neuropathy, learning disabilities, and short stature. HP is less commonly observed.

The mutation patterns of mtDNA are complex, influenced not only by inheritance but also by non-genetic factors, such as age (7). mtDNA mutations are highly heterogeneous, and the mutation burden can vary significantly across different tissue cells in the same individual. Disease onset typically occurs once the mutation burden reaches a certain threshold, which differs across various mutation sites. For instance, the mutation thresholds for the thirteen protein-coding genes in mtDNA are relatively high, while those for tRNA genes are lower. Some mutations can cause disease even when the proportion of mutant mtDNA is below 20%. Research by Ciafaloni et al. has shown that the proportion of mutant genomes in MELAS patients is significantly higher than in their asymptomatic relatives (8).

In this case, only the child and his mother exhibited the m.3243A > G mutation, while his older brother and sister were wild-type. According to our high-throughput sequencing results, the heteroplasmy level of the m.3243A > G mutation in the child's peripheral blood was 86.7%, compared to just 4.8% in the mother. This explains why the child manifested clinical symptoms, while the family members remained asymptomatic. However, given the low heteroplasmy level in the mother's sample, orthogonal validation of the high-throughput sequencing results using a more sensitive method, such as digital PCR, was warranted. Unfortunately, due to resource constraints, this additional validation was not feasible.

In adults, HP is primarily observed following thyroid surgery in clinical practice. However, in children, HP is more commonly idiopathic or hereditary, as seen in conditions such as 22q11.2 microdeletion syndrome, HP-deafness-renal dysplasia (HDR) syndrome, and Kenny-Caffey syndrome. Mutations in multiple genes, including AIRE, GATA3, CASR, GNA11, and PTH, have been identified as potential causes of HP in children (9). It can also occur as part of mitochondrial diseases, such as Kearns-Sayre syndrome (KSS) or MELAS syndrome. In mitochondrial diseases, patients with mtDNA duplications or deletions have a higher incidence of endocrine disorders. This explains why HP is more commonly associated with KSS, whereas its occurrence in MELAS patients with mtDNA point mutations is rarer (10).

The exact mechanism by which mitochondrial diseases lead to HP remains unclear, but it may involve the disruption of proteins in the mitochondrial oxidative respiratory chain, leading to ATP synthesis disorders and insufficient PTH production or secretion, ultimately resulting in HP. In this case, the child's tetany may be attributed to multiple factors. Although the precise mechanisms behind epileptic-like seizures in MELAS patients remain incompletely understood, certain studies propose that they may stem from an inadequate energy supply and a deficiency of nitric oxide (NO). In MELAS, the mitochondrial oxidative phosphorylation process is disrupted, preventing the production of adequate ATP to meet energy demands. Additionally, lactic acid cannot be fully utilized, leading to a metabolic imbalance in neurons. This, in turn, impairs normal neuronal function and contributes to damage in the cerebral cortex, triggering epileptic-like seizures. In addition to disturbances in energy metabolism, MELAS patients often face NO deficiency. NO, produced by vascular endothelial cells, plays a crucial role in relaxing vascular smooth muscle and maintaining the patency of small blood vessels. The reduced availability of NO precursors, such as arginine and citrulline, in MELAS patients affects NO production, leading to symptoms like hypoxemia, seizures, and stroke-like episodes (11). Supplementation with the NO precursor arginine has been shown to prolong the interval between seizures, reduce the frequency and severity of seizures, and significantly improve patient survival (12).

Additionally, basal ganglia calcification (BGC) is observed in 73.8% of patients with idiopathic HP and in 60% of MELAS patients. While BGC itself is unlikely to directly cause seizures, with an incidence of epilepsy of only 0.90% in sporadic BGC (13), intracranial calcification in HP has been identified as an independent risk factor for epileptic-like seizures (14). Furthermore, calcium-phosphorus metabolism disturbances in HP can contribute to tetany. Electroencephalographic patterns during seizures in hypoparathyroid patients resemble those in patients with epilepsy, and antiepileptic drugs can temporarily control tetany, often leading to misdiagnosis of seizures. This is likely due to the increased neuronal excitability resulting from low extracellular calcium concentrations (15). Managing the calcium-phosphorus ratio can not only reduce tetany episodes but also help delay the progression of BGC, which is critical for the child's prognosis.

In this case, following treatment for seizure control and metabolic improvement, there was a significant reduction in cerebral cortical injury. Thus, we believe that improving metabolism and controlling epileptic seizures are key components of effective MELAS treatment. The treatment of MELAS requires a multidisciplinary approach. Currently, no specific cure exists for MELAS. The primary treatment strategies focus on controlling seizures, improving calcium-phosphorus metabolism, and managing hyperlactatemia. As endocrinologists, when encountering patients with HP, it is important to be vigilant for the possibility of coexisting mitochondrial diseases. For patients presenting with lactic acidosis and basal ganglia calcification, MELAS should be strongly suspected. Early genetic testing is essential for an accurate diagnosis and can have a significant positive impact on patient prognosis.

ijem-23-2-161585-s001.pdf

## Data Availability

The dataset presented in the study is available on request from the corresponding author during submission or after publication. The data are not publicly available due to involving patient privacy.
