# Supplementary material for: Hypoparathyroidism in a Child with MELAS Syndrome: A Case Report of Severe Lactic Acidosis and Symmetrical Bilateral Basal Ganglia Calcification
Source: Int J Endocrinol Metab. 2025 Apr 30;23(2):e161585. doi: 10.5812/ijem-161585 (PMC12296682; doi:10.5812/ijem-161585)
Supplement: ijem-23-2-161585-s001.pdf [file ijem-23-2-161585-s001.pdf]

Appendix **1.** Changes in the laboratory indicators of the child across each examination.

	baseline	After the treatment	The first discharge	the second hospitalization	The second discharge
lactic acid(mmol/L)	6.0	4.8	3.5	6.6	3.5
25OH-VitD(ng/mL)	7.8	27.3	*	33.9	/
PTH(pg/mL)	6.62	4.59	<3.00	<3.00	<3.00
serum calcium(mmol/L)	0.97	0.98	1.05	1.19	1.20
serum phosphorus(mmol/L)	1.47	1.86	1.55	1.65	1.91
urinary calcium(mmol/L)	0.1	3.58	*		
urinary phosphorus(mmol/L)	4.8	7.10	*		
urinary calcium(mmol/24h)	0.15	2.67	*		
urinary phosphorus(mmol/24h)	7.34	5.29	*		

*：The results after treatment represent the findings at the time of the first discharge.
